# Insights into the interactions between tetracycline, its degradation products and bovine serum albumin

**DOI:** 10.1186/s40064-016-2349-4

**Published:** 2016-07-13

**Authors:** Xingyu Tong, Manfei Mao, Jingqian Xie, Kefeng Zhang, Dongmei Xu

**Affiliations:** College of Biology and Environmental Engineering, Zhejiang Shuren University, Hangzhou, 310015 China; Institute of Environmental Science, Zhejiang University, Hangzhou, 310058 China

**Keywords:** Tetracycline, Degradation products, BSA, Spectroscopic method, Molecular docking

## Abstract

Tetracyclines (TCs) are the most widely used antibiotics in the world. Because antibiotics have low bioavailability and are difficult to completely remove using current sewage treatment facilities, residual TCs and their degradation products in the environment, animal and plant foodstuffs and personal care products may enter the body through the food chain, thus causing unpredictable effects on human health. We studied bovine serum albumin (BSA) (a functional protein) as a target of tetracycline-induced toxicity by examining its interactions with TC, anhydrotetracycline (ATC) and epitetracycline (ETC), based on a fluorescence spectroscopy and molecular docking method under simulated physiological conditions. The interaction mechanism was elucidated at the molecular level. The results show that TC, ATC and ETC bind at site II of BSA and interact mainly through hydrogen bonding interactions and van der Waals interactions. The binding affinities can be ranked in the order ATC > TC > ETC.

## Background

As one of the most important medical findings of the twentieth century, antibiotics not only make great contributions to the treatment of human and animal bacterial infections, but have been used globally at subclinical doses in the animal breeding industry as feed additives for a long time (Gu and Karthikeyan 2005; Hvistendahl 2012). Specifically, tetracyclines (TCs) are a large group of antibiotics widespread used in human and veterinary medicine and account for approximately 29 % of total antibiotic use (Khetan and Collins 2007; Wammer et al. 2011). TCs are broad-spectrum antibiotics synthesized or semi-synthesized from actinomycetes, and are widely used in livestock and poultry breeding and aquaculture as veterinary and feed additives (Bowman et al. 2011), ranking second in the global production and application of veterinary drugs. Generally, TCs cannot be completely absorbed and metabolized after ingestion by animals but are excreted in feces and urine as prototype and active metabolites (Hu et al. 2011). Because antibiotics also cannot be completely removed through existing sewage treatment processes, they ultimately reach the environment and accumulate through surface runoff scour percolation, effluents from sewage treatment plants and the deposition on land of manure from livestock and poultry (Spongberg and Witter 2008). TCs have now been detected in many countries and in various environmental systems, including surface water (Kim and Carlson 2007), ground water (Krapac et al. 2005) and soil (Kulshrestha et al. 2004), as well as in animals and plants (Ji et al. 2010).

TCs entering the environment can produce corresponding metabolites by epimerism, dehydration, proton transfer and other methods under the actions of environmental biological and non-biological factors (Jia et al. 2009). Mackie et al. (2006) detected dehydrated degradation products of tetracycline, chlortetracycline and oxytetracycline in the ground water under soil that had been subjected to long-term fertilization with manure. Residues in honey of TCs used to prevent foulbrood infection in bees readily degrade to epimers during processing and storage. Liu et al. (2011) verified the existence of epi-degradation products from tetracycline and oxytetracycline (at 4.9 and 3.8 µg kg^−1^, respectively). Dehydration products of TCs can be detected in personal care products such as facial cleansers and bath foams. TC residues and their catabolites that accumulate in the environment, animal- and plant-based foods and personal care products are likely to be transferred into the human body through the food chain, resulting in negative impacts on human health. Current research shows that catabolites of TCs usually have relatively decreased activities but possibly increased toxicities than the parent compound (Halling-Sørensen et al. 2002). The epimers of TCs can cause strong toxic effects clinically in animals and human beings (Daghrir and Drogui 2013). Halling-Sørensen et al. (2002) also found that the degradation products of some TCs had decreased activities but stronger toxic effects than their parent antibiotics towards drug-resistance bacteria and soil bacteria in the environment.

Serum albumin, the most abundant protein in plasma, has important carrier functionality, is one of the components of the blood buffer system and is the major component responsible for maintaining colloidal osmotic pressure in blood. Its ability to combine with various endogenous and exogenous pollutants also makes it important in maintaining the free activity concentration of such substances in plasma and affects their transport, distribution, storage and metabolic processes inside the organism. The interactions between TCs and biomacromolecules such as serum proteins have been reported in the literature (Chi and Liu 2011; Khan et al. 2002), but research into the influence of TC degradation products on macromolecular structures and functions has been rarely reported to date. Based on fluorescence spectroscopy and molecular docking technology under simulated physiological conditions, this paper focuses on interactions of tetracycline (TC) and its degradation products anhydrotetracycline (ATC) and epitetracycline (ETC) with serum albumin. In particular, we examine the effects of such substances on protein conformation and the modes of action between the three drugs and serum protein from the molecular perspective. The results provide insights into the processes of distribution, transfer and transportation of TC and its degradation products to toxic endpoints in the human body and associated health risks. In addition, the results provide data regarding safe dosages and risk assessment for such substances.

## Methods

**Fig. 1:**
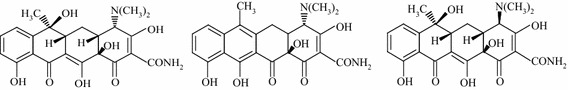
Structure of TC, ATC and ETC

Main reagents: analytically pure reagents were purchased, including bovine serum albumin (BSA, Sigma Chemicals, St. Louis, MO, USA), tetracycline hydrochloride (Shanghai Sangon Biotechnology Co., Ltd., Shanghai, China, imported and sub-packed with 98.5 % of purity), anhydrotetracycline and epitetracycline hydrochloride (Acros Organics Co., Ltd., Geel, Belgium, with 100 % purity, structures as shown in Fig. 1), Tris, hydrochloric acid (Hua Dong Medicine Co., Ltd., Hangzhou, China). Double-distilled water was used in all tests.

One milliliter of Tris–HCl buffer solution at 0.2 mol L^−1^ and pH 7.40, 1 mL of sodium chloride solution at 1 mol L^−1^ and 1 mL of standard BSA solution at 2.0 × 10^−5^ mol L^−1^ were added in sequence into 10 mL colorimetric tubes. These were then gently mixed and held for 10 min. Using a micro syringe, a certain volume of drug was added to each colorimetric tube and diluted with distilled water to 10 mL. The final concentrations were 0, 0.2, 0.4, 0.8, 1.2, 1.6, 2.0, 2.4, 2.8, 3.2, 3.6 and 4.0 × 10^−5^ mol L^−1^ respectively. All solutions were then placed at constant temperatures of 293, 298, 304 and 310 K for 60 min until binding equilibrium was reached. The fluorospectrophotometer F-4600 was then used to measure the emission spectra over the wavelength range 300–400 nm of the test solutions after obtaining reaction equilibrium at constant temperature. The maximum emission wavelength and fluorescence intensity were recorded. The excitation wavelength was 285 nm, the slit width was 5 nm and the scanning speed was 240 nm min^−1^. The synchronous fluorescence spectra of sample solutions were scanned over the wavelength range 300–400 nm under ∆λ = 15 nm and ∆λ = 60 nm constant steps.

To define the type of quenching, it was initially assumed to be dynamic quenching, and the fluorescent data were then analyzed using the Stem–Volmer equation.

For static quenching, when the ligand micromolecule combines with the specific site of a biomacromolecule, the binding constant (K_a_) and binding-site number (n) can be calculated using the following formula:

1 $$\lg\frac{F_{0} - F}{F} = \lg KA + n\lg [Q]$$

In Eq. (1), K_A_ represents the binding constant, n represents the number of micromolecules and protein binding-site, which is actually the concentration of free micromolecules because the concentration of micromolecules is lower than that of the protein and the combination through non-covalent bonds is relatively weak. Thus, we regard [Q] as the total concentration of micromolecules.

When the temperature does not vary markedly, the reaction enthalpies (Δ*H*) can be regarded as constant, and the enthalpy and entropy changes of reaction can be calculated using the van’t Hoff equation. The free energy change can be further calculated using the formula Δ*G* = Δ*H* − TΔ*S*.

The crystal structure of BSA in the system BSA-TC/ATC/ETC was downloaded from the Protein Database (PDB ID: 4JK4, 2.65 Å). Water molecules were removed, nonpolar hydrogen atoms were merged, and rotatable bonds were also defined (Chi and Liu 2011). The MOE 2009 software (Chemical Computation Group, Montreal, Quebec, Canada) was applied to build and optimize the tertiary structures of these three small molecules at minimal energy. Molecular docking was performed using MVD 4.0 software. The binding pocket was designated as a sphere with radius 20 Å around the ligands. Thirty runs were carried out, producing thirty conformations for selection. The poses which that had the highest scores were chosen for the molecular dockings.

## Results and discussion

**Fig. 2:**
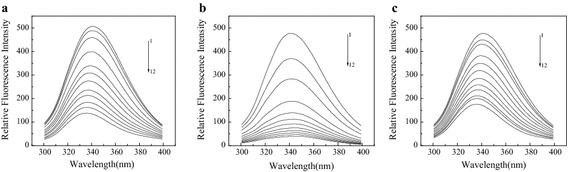
Effect of TC (**a**)/ATC (**b**)/ETC (**c**) on fluorescence spectra of BSA. Conditions: C_BSA_ = 2.0 × 10^−6^ mol L^−1^; λ_ex_ = 285 nm; T = 298 K; pH = 7.40; C from 1–12: 0, 0.2, 0.4, 0.8, 1.2, 1.6, 2.0, 2.4, 2.8, 3.2, 3.6 and 4.0 (×10^−5^ mol L^−1^)

It can be seen from Fig. 2, from the influences of TC and its degradation products ATC, ETC on the BSA fluorescence spectrum, that all these three drugs have significant quenching effects on BSA intrinsic fluorescence. In terms of shifting the maximum emission wavelength, ATC has the most obvious quenching effects amongst the three compounds. Under the actions of TC and ETC, the maximum emission wavelength of BSA underwent a blue shift, indicating that TC and ETC enhance the hydrophobicity of the fluorophore microenvironment of BSA (i.e. affecting amino acid residues with aromatic nuclei) (Yuan et al. 1998). In contrast, the action of ATC enhanced the polarity of the fluorophore microenvironment of BSA and decreased its hydrophobicity. Thus, the maximum emission wavelength of the ATC-BSA system underwent a red shift from 341.4 to 346.4 nm, consistent with the result of Burgos et al. (2011), indicating that there is an elimination reaction of a water molecule between the alcohol hydroxyl of C6 and the hydrogen of C5a, leading to a difference in the TC molecular structure. Thus, the effects of ATC, TC, and ETC on the BSA fluorescence chromophore microenvironment were different.

**Fig. 3:**
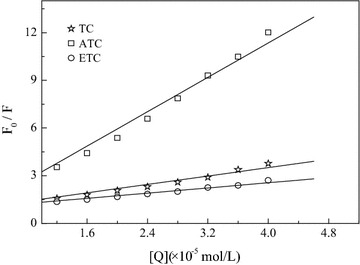
Stern–Volmer curves of TC and its degradation products on fluorescence intensity of BSA

Fluorescence quenching comprises both dynamic and static quenching. Identifying the type of fluorescence quenching caused by TC and its degradation products, can confirm whether or not the drugs and BSA can form complexes. The Stern–Volmer curve (298 K) of BSA fluorescence quenching caused by the three drugs is shown in Fig. 3. Generally, the fluorescent lifetime of biomacromolecules is 10^−8^ s, and thus, the BSA quenching rate constant caused by TC at different temperatures and its degradation products ATC and ETC can be calculated. The results are shown in Table 1.

**Table 1:** Fluorescence quenching constants of TC and its degradation products on BSA

Drug	Temperature (K)	K_q_ (L mol s^−1^)	K_sv_ (L mol^−1^)	R^2^
TC	293	6.61 × 10^12^	6.61 × 10^4^	0.989
	298	6.27 × 10^12^	6.27 × 10^4^	0.981
	304	6.05 × 10^12^	6.05 × 10^4^	0.989
	310	5.70 × 10^12^	5.70 × 10^4^	0.990
ATC	293	2.70 × 10^13^	2.70 × 10^5^	0.994
	298	2.61 × 10^13^	2.61 × 10^5^	0.993
	304	2.36 × 10^13^	2.36 × 10^5^	0.995
	310	2.10 × 10^13^	2.10 × 10^5^	0.992
ETC	293	4.31 × 10^12^	4.31 × 10^4^	0.996
	298	4.09 × 10^12^	4.09 × 10^4^	0.992
	304	3.71 × 10^12^	3.71 × 10^4^	0.993
	310	3.49 × 10^12^	3.49 × 10^4^	0.999

It can be seen from the data in Table 1 that the quenching constant K decreased with increasing temperature, and the order of magnitude of Kq reached 10^12^, which is 2–3 orders of magnitude higher than the maximum diffusion and collisional quenching constant (2 × 10^10^ L mol^−1^ s^−1^) of biomacromolecules in aqueous solution (Pan et al. 2011).

The BSA fluorescence quenching caused by the three drugs were identified as static quenching, so the binding constant and binding-site number can be calculated using Eq. (1). The results are shown in Table 2. The binding-site numbers “n” for TC, ATC, ETC and BSA were all about 1, which indicates that TC and its degradation products use one binding site to interact with BSA and form complexes. At the same time, based on the binding constants, it can be seen that the combinations between these three drugs and BSA were quite strong. Table 2 shows that at 298 K, the binding constants between TC, ATC, ETC and BSA were, respectively, 4.50 × 10^4^, 1.45 × 10^5^ and 2.44 × 10^4^ L mol^−1^. The sequence of binding affinity was therefore: ATC > TC > ETC. Zhang et al. (2006) found that the Microtox acute toxicity of the TC parent on *Vibrio fischeri* was stronger than ETC. This was because, after the epimerization reaction, the dimethylamino of C4 caused steric hindrance, which inhibits the drug from combining with the end of the 30 s ribosomal subunit to a great extent.

**Table 2:** Binding constants, binding-site numbers and relative thermodynamic parameters of TC and its degradation products with BSA

Drug	Temperature (K)	K_A_ (L mol^−1^)	n	R^2^	∆*G* (KJ mol^−1^)	∆*H* (KJ mol^−1^)	∆*S* (J mol^−1^ K^−1^)
TC	293	5.13 × 10^4^	1.01	0.996	−26.40	−19.41	23.86
	298	4.50 × 10^4^	1.17	0.997	−26.52		
	304	3.60 × 10^4^	1.17	0.999	−26.66		
	310	3.39 × 10^4^	1.18	0.999	−26.81		
ATC	293	1.87 × 10^5^	1.07	0.999	−29.40	−15.10	48.80
	298	1.45 × 10^5^	1.18	0.998	−29.64		
	304	1.36 × 10^5^	1.09	0.998	−29.94		
	310	1.30 × 10^5^	1.08	0.999	−30.23		
ETC	293	2.95 × 10^4^	1.00	0.998	−24.96	−12.12	43.81
	298	2.44 × 10^4^	1.05	0.999	−25.18		
	304	2.30 × 10^4^	1.12	0.999	**−**25.44		
	310	2.21 × 10^4^	1.17	0.995	−25.70		

**Fig. 4:**
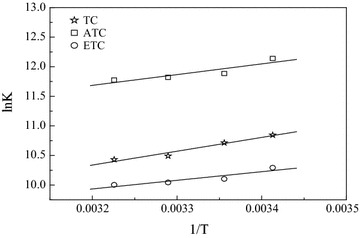
The Van’t Hoff curves

Figure 4 was drawn based on the reciprocal of binding constant and temperature according to van’t Hoff equation to calculate the values of ∆H, ∆S and ∆G according to the intercept and slope of the curves (Table 2). It can be seen from the data in the table, that the reaction system free energies, Δ*G*, for the three drugs were all negative, indicating that the combination of TC and its degradation products ATC, ETC with BSA are spontaneous reaction processes. In addition, the ∆H had negative values while the ∆S was positive in the three systems. From this observation, based on the theory regarding the nature of the binding forces between biomacromolecules and biomicromolecules proposed by Ross and others, it can be estimated that hydrogen-bond and electrostatic interaction had the greatest contributions during interactions between tetracycline, its degradation products and BSA (Ross and Subramanian 1981; Zhang et al. 2013).

**Fig. 5:**
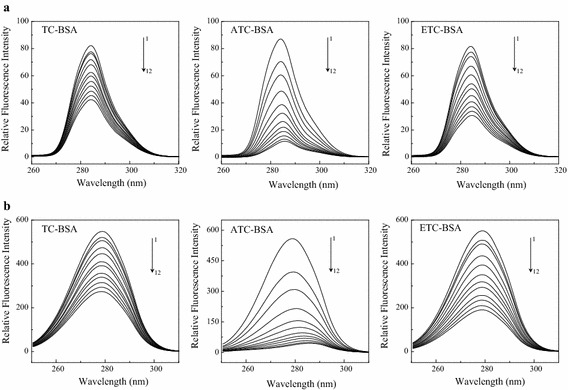
Synchronous fluorescence spectra of TC and its degradation products on BSA. Conditions: CBSA = 2.0 × 10^−6^ mol L^−1^; pH = 7.40; T = 298 K; **a** Δλ = 15 nm; **b** Δλ = 60 nm; C from 1–12: 0, 0.2, 0.4, 0.8, 1.2, 1.6, 2.0, 2.4, 2.8, 3.2, 3.6 and 4.0 (×10^−5^ mol L^−1^)

When the wavelength differential (∆λ) of synchronous fluorescence spectroscopy is 15 or 60 nm, the characteristic spectra of tyrosine and tryptophan residues, respectively can be obtained (Wang et al. 2007). A change in the synchronous fluorescence peak position can indicate changes in the microenvironment of amino acid residues, which further indicates changes in the protein conformation (Vekshin 1996). BSA synchronous fluorescence curves under the actions of TC, ATC, ETC are shown in Fig. 5. It can be seen from Fig. 5 that the peak position of the characteristic fluorescence spectra under the actions of TC and ETC, which reflects the microenvironment of tyrosine residues, did not change markedly, but the peak positions in the characteristic fluorescence spectra for tryptophan residues had blue shifts from 279.0 to 278.4 and 278.6 nm, respectively. This indicates that the action of TC, ETC enhanced the hydrophobicity and decreased the polarity of the microenvironment of the tryptophan residues but did not affect the microenvironment of the tyrosine residues. In contrast, in the presence of ATC, the maximum emission wavelengths of BSA tyrosine, tryptophan residues had red shifts from 284 to 285.8 nm and from 279 to 286 nm, respectively, indicating that ATC decreased the hydrophobicity and enhanced the polarity of the tyrosine and tryptophan residues.

**Fig. 6:**
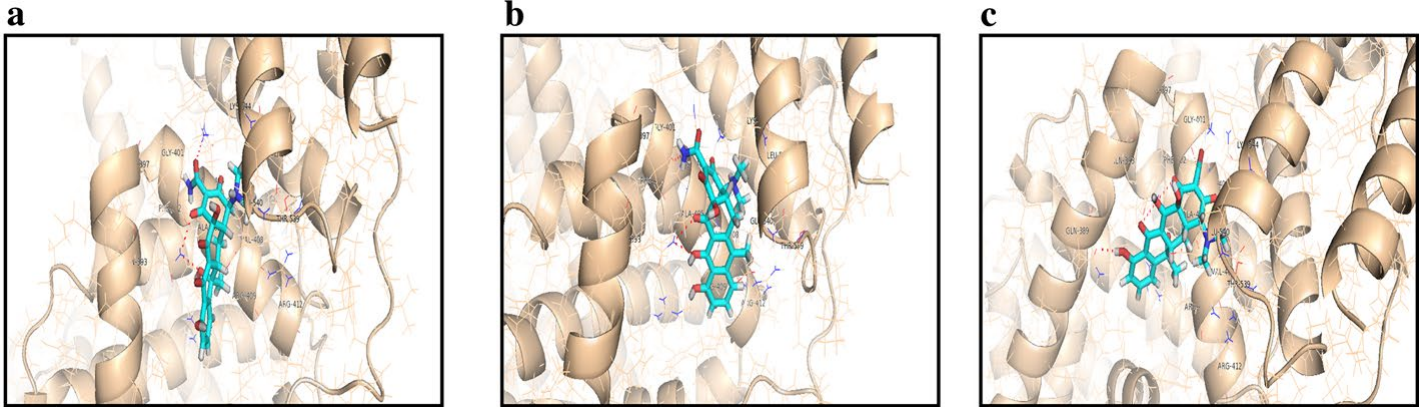
The combining model of TC (**a**)/ATC (**b**)/ETC (**c**) with BSA

TC and its degradation products ATC, ETC were automatically docked into site II (IIIA) on BSA. Four hydrogen bonds were formed between BSA and TC/ATC/ETC. The interaction energies and the bond lengths between TC/ATE/ETC and amino residues are listed in Table 3 and the combined model is shown in Fig. 6. ATC and oxygen atoms could form two hydrogen bonds with Gly401, Leu543, while just one H–O hydrogen bond occurred for TC with Gly 401 and ETC with Gly 401. Moreover, the value of the calculated free energy of binding (ΔG) of TC was smaller than that for ETC. These confirmed that the strengths of interactions between TC/ATC/ETC and BSA were in the order ATC > TC > ETC. In addition, TC was mainly surrounded by hydrophilic residues: Asn 404, and Thr 539, and electrically charged residues: Arg 409, Arg 412, Glu540, and Lys544. Electrically charged residues Arg 409, Arg 412, Glu 540, Lys 544 Arg 412 were found close to ATC, while ETC also interfaced with a charged area comprising Arg412, Lys544. Thus, the results above confirm that hydrogen-bond and electrostatic forces played significant roles in the interactions, consistent with the results of the thermodynamic analysis described above.

**Table 3:** Interaction energies between TC/ATC/ETC and responsive amino acid residues in molecular docking

System	Amino residues	Interaction energy (kJ mol^−1^)	Length (Å)
TC	Gln 393	−2.34	2.58
		−2.13	3.17
	Gly 401	−0.39	2.45
	Lys 544	−1.36	3.33
ATC	Gln 393	−1.87	2.52
		−2.50	2.72
	Gly 401	−0.64	2.69
	Leu 543	−2.50	3.08
ETC	Gln393	−2.50	2.62
	Gly 401	−2.50	2.86
		−1.51	3.02
	Lys 544	−2.50	2.97

## Conclusions

TC and its degradation products (ATC and ETC) interact with BSA. The binding affinities were in the order ATC > TC > ETC. The drugs bind mainly at site II of BSA and the three drugs interact with BSA mainly through hydrogen bonding and van der Waals interactions. TC and ETC enhance the hydrophobicity of the microenvironment of BSA tryptophan residues. In contrast, the action of ATC enhanced the polarity of the fluorophore microenvironment of BSA tryptophan and tyrosine residues and decreased its hydrophobicity. The ATC has a stronger affinity towards BSA, tending to cause more significant conformational changes in BSA.
